# Impact of surgical treatment of pectus carinatum on cardiopulmonary function: a prospective study

**DOI:** 10.1093/ejcts/ezaa335

**Published:** 2020-11-19

**Authors:** Barbara Del Frari, Stephan Sigl, Anton H Schwabegger, Cornelia Blank, David Morawetz, Eva Gassner, Wolfgang Schobersberger

**Affiliations:** 1 Department of Plastic, Reconstructive and Aesthetic Surgery, Medical University Innsbruck, Innsbruck, Austria; 2 Department of Psychology and Medical Sciences, Institute of Sports Medicine, Alpine Medicine & Health Tourism (ISAG), University for Health Sciences, Medical Informatics and Technology (UMIT), Hall in Tyrol, Austria; 3 Institute for Sports Medicine, Alpine Medicine & Health Tourism (ISAG), Tirol Kliniken GmbH Innsbruck, Innsbruck, Austria; 4 Department of Radiology, Medical University Innsbruck, Innsbruck, Austria

**Keywords:** Pectus carinatum, Thoracoplasty, Cardiopulmonary function, Spiroergometry, Spirometry

## Abstract

**OBJECTIVES:**

The frequency of sternochondroplasty in cases of pectus carinatum (PC) has increased due to greater surgeon experience and modified surgical techniques. PC deformity does not usually cause cardiopulmonary malfunction or impairment. However, whether cardiopulmonary function changes after surgical repair remains a matter of controversy. The aim of our prospective study was to determine if surgery changes preoperative cardiopulmonary function.

**METHODS:**

Nineteen patients (16 males, 3 females) were enrolled in a prospective, open-label, single-arm, single-centre clinical trial (Impact of Surgical Treatments of Thoracic Deformation on Cardiopulmonary Function) (NCT02163265) between July 2013 and January 2017. All patients underwent PC repair via a modified Ravitch procedure and wore a lightweight, patient-controlled chest brace for 8 weeks postoperatively (the Innsbruck protocol). The average follow-up surgical examination was 8.3 months after surgery. In all enrolled patients, before surgery and not before 6 months postoperatively chest X-ray, 3-dimensional volume-rendered computed tomography thorax imaging, cardiopulmonary function tests with stepwise cycle spiroergometry (sitting and supine position) and Doppler echocardiography were performed; questionnaires about daily physical activity were also completed.

**RESULTS:**

Fourteen patients (aged 16.3 ± 2.6 years at study entry) completed the study. Changes in submaximal and peak power output were not detected during sitting, or when in the supine position. Also, no clinically relevant postoperative changes in spirometry or echocardiography were noted.

**CONCLUSIONS:**

Our findings confirm that surgical correction of PC does not impair cardiopulmonary function at rest or during physical exercise.

**Clinical registration number:**

clinicaltrials.gov NCT02163265.

**Figure:**
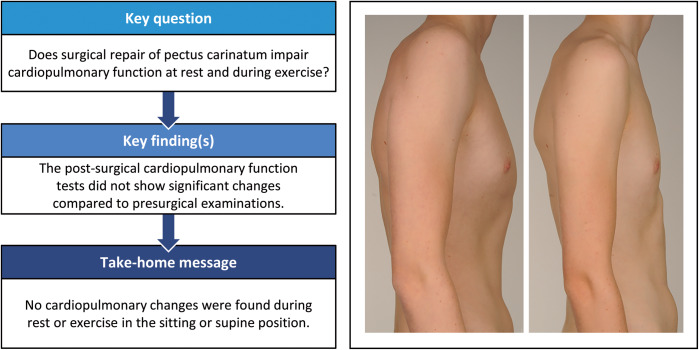

## INTRODUCTION

Pectus carinatum (PC), or keel chest deformity, refers to protrusion of the sternum possibly due to the overgrowth of costal cartilages and the adjacent ribs, and/or asymmetric cartilages [1–4]. PC is the second most common congenital anterior chest wall deformity [3]. The overall prevalence of PC is 0.6%, and it is more common in men. Symptoms in most patients are vague and may include retarded growth, exertional or chronic dyspnoea, asthmatic attacks and palpitations [5]. Furthermore, patients may suffer from a poor self-image and a lower quality of life due to cosmetic issues [6].

For decades, surgical correction of PC was performed primarily for cosmetic and psychological indications, without proven documentation of any improvements or impairments in physiological parameters. The traditional surgical repair technique has involved resection of the abnormal costal cartilages and sternal osteotomy, with or without strut placement [1, 7]. Several modifications in the surgical technique for correction of PC have since been reported [1, 8–11]. Although PC correction is carried out more frequently today, scientific evidence regarding the effects thereof on cardiopulmonary function is still lacking [12]. A systematic review of preoperative and postoperative cardiopulmonary function revealed small case numbers and heterogeneity in the examinations applied [12–16]. Only 5 clinical studies, with the largest series reporting only 5 PC patients, included post-surgical cardiopulmonary function tests [12, 13]. Thus, it remains unclear if surgical correction on PC has no or even negative effects on cardiopulmonary function at rest and during exhaustive exercise. Therefore, the aim of this prospective study was to determine if surgical intervention for PC has any effect on postoperative cardiopulmonary function at rest and during exercise in a larger cohort with higher statistical power. To the best of our knowledge, a prospective study including only PC patients utilizing pre- and postoperative comparison of cardiopulmonary function and exercise has not been published previously.

## PATIENTS AND METHODS

### Study design

Patients with diagnosed PC from the Department of Plastic, Reconstructive and Aesthetic Surgery of the Medical University (Innsbruck, Austria) were invited to participate in this non-randomized, prospective, open-label, single-arm, single-centre feasibility study. The study was approved by the Institutional Review Board of Medical University Innsbruck (Approval Number AN4741) and registered at clinicaltrials.gov (ClinicalTrials.gov number: NCT02163265). The study inclusion criteria were (i) patient age between 10 and 50 years; (ii) male or female with PC deformities; and (iii) minor to severe PC. These age limits were proposed by the Ethics Committee. In addition, our patient selection criteria were by the previously made clinical decisions to operate only on PC patients from age at least 14 years (due to body maturation), but not older than 20 years (due to public health insurance restrictions). The exclusion criteria were (i) Poland’s syndrome; (ii) previous repair of PC using any technique; (iii) previous thoracic surgery; (iv) congenital heart disease; (v) history of major anaesthetic risk factors such as malignant hyperthermia or pregnancy; and (vi) standard exclusions for cycle spiroergometry.

Patients provided informed consent before being enrolled in the study between July 2013 and January 2017. Baseline characteristics including demographic data, medical history, calliper measurement of the transversal and sagittal chest diameters (MedXpert Company GmbH, Heitersheim, Germany) and body mass index were obtained; standardized photographic documentation was also collected. Patients deemed as surgical candidates were screened using a standardized protocol for pre- and 6-month postoperative chest X-ray and 3-dimensional (3D) volume-rendered computed tomography (CT) thorax imaging. The Haller Index (HI), as the most widely used measure of severity and the anatomic effectiveness of surgical repair, was calculated [17]. Cardiopulmonary function tests (spirometry, electrocardiography, transthoracic Doppler-echocardiography at rest followed by cycle spiroergometry in the sitting and supine positions until exhaustion) were performed in all patients, preoperatively and not before 6 months postoperatively. Physical activity during daily life was evaluated with standardized questionnaires.

During the study period between July 2013 and January 2017, 19 patients (16 males, 3 females) met the inclusion criteria and were evaluated in terms of their suitability for surgical treatment. Five patients were excluded during the study due to missing follow-up data (2 males) or because they did not request further surgical treatment after the initial diagnostic assessments (1 male, 2 females). Except for mild scoliosis (*n* = 4), no other concomitant morbidities were documented.

**Figure 1:**
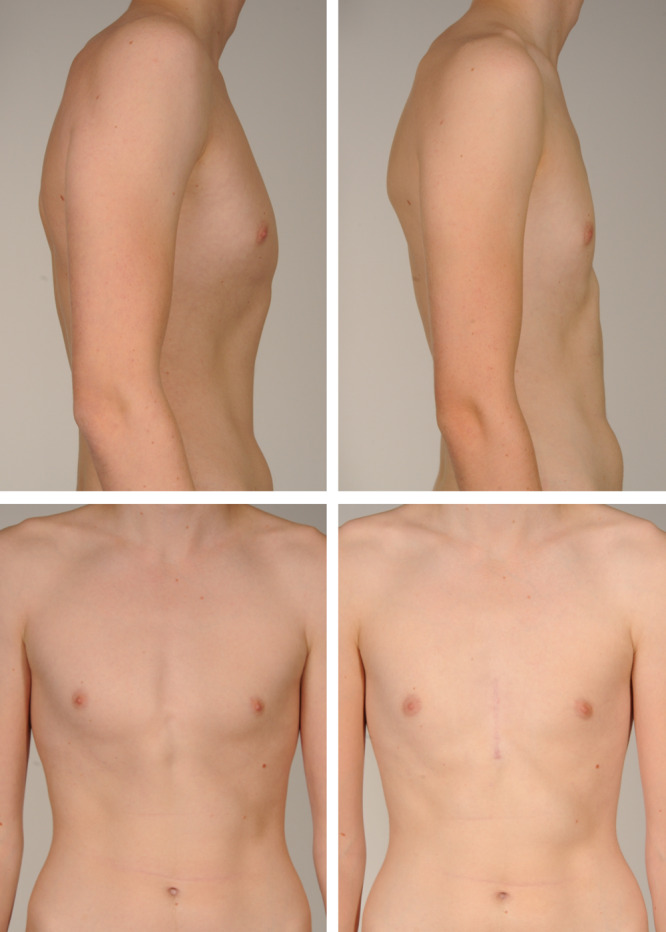
Clinical presentation of a male patient (18 years of age), presurgery (left picture) and at 6 months post-surgery (right picture), who underwent parasternal resection of costal cartilages V–VII of the right hemithorax and V–VII of the left hemithorax.

The surgical repair was in accordance with a previously described technique and was performed by a single surgeon [11]. Throughout the study period, the patients underwent serial clinical examinations (Fig. 1).

### Surgical technique

In the supine position, a presternal midline vertical incision was performed in men, whereas in women the incision was made along the inframammary fold (unilaterally or bilaterally). Skin flaps were mobilized and the parasternal area of the pectoralis major muscle was exposed. The muscle was split along the direction of its fibres just above the protruding rib cartilage [10, 11]. The perichondrium was incised and subperichondrial costal-cartilage resection was performed. A transverse sternotomy was performed across the ventral lamella of the anterior sternum at the appropriate level, and the distal sternum was pressed to the desired position. To stabilize the result, the empty perichondrium tubes were shortened by reefing sutures. After wound suture, a circumferential adhesive dressing was applied for 48 h, followed by a custom-made, lightweight aluminium keel chest brace with silicone padding to ensure stability*.* Patients wore the brace for at least 23 h per day, for a period of 8 weeks (the Innsbruck protocol) [11].

### Testing equipment

#### Pulmonary function test

The pulmonary function was assessed by spirometry (GE Medical Systems IT Inc., Milwaukee, WI, USA). Parameters of interest included the forced vital capacity (FVC; l), forced expiratory volume in 1 s (FEV1; l) and the Tiffeneau Index (FEV1/FVC; %). To exclude any possible influences of growth in the adolescent patients, these parameters were also calculated as percentage relative to an age-adjusted reference population [data given as percent predicted values (% pred) according to Quanjer *et al.* [18]].

#### Echocardiography

Transthoracic echocardiography (2-dimensional, M-mode) was recorded to exclude potential mitral valve regurgitation and other possible structural abnormalities, as well as to determine fractional shortening, left ventricular systolic and diastolic diameter, ejection fraction and right ventricular diastolic diameter (ACUSON SC2000; Siemens, Munich, Germany).

#### Cycle spiroergometry

Patients performed stepwise cycle ergometry in the sitting (Lode B.V., Groningen, Netherlands) and supine positions (ebike L; Ergoline GmbH, Bitz, Germany), until exhaustion or objective criteria for exercise termination were met [19]. Oxygen consumption (VO_2_) was measured via gas analyses (Care Fusion; Vyntus CPX, Hoechberg, Germany). The test started at 25 W, with the workload increasing by 25 W every 120 s. The same protocol was used for both spiroergometries, similar to the recommendations of Malek and Coburn [20]. Capillary blood (20 µl) for lactate analysis was collected from the hyperaemic ear lobe every 120 s at the end of each workload step (Biosen S-Line Lab+; EKF Diagnostic, Barleben, Germany). The parameters of interest were the relative mean power at 2 and 4 mmol/l lactate (W/kg), peak power (absolute and relative), heart rate (peak), VO_2_ peak ml/kg/min and peak blood lactate concentration.

### Computed tomography examination

**Figure 2:**
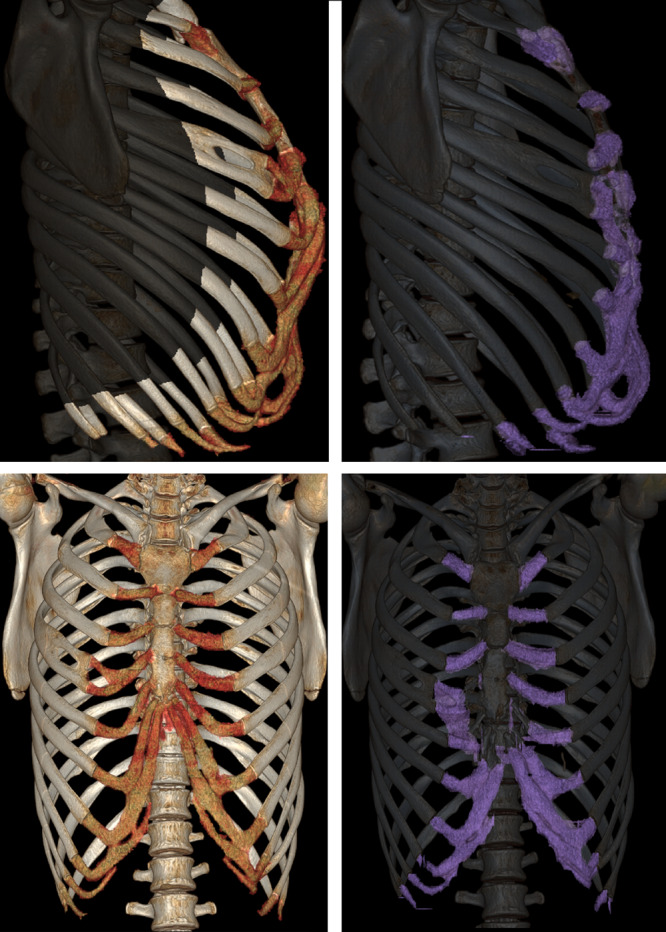
Three-dimensional volume-rendered computed tomography thorax imaging of the same patient as in Fig. 1, presurgery (left) and at 6 months post-surgery (right).

CT scans were acquired using a 64-slice multidetector computed tomography instrument (image reconstruction, 0.625 and 2.5 mm; coronal/sagittal reformations, 3 mm). 3D volume-rendered images were generated with the segmentation of bone and cartilage to build a colour-coded 3D model of the thoracic cage (Fig. 2). The radiation dose (CT dose index) ranged from 3.5 to 6.5 mSv. The HI was calculated by dividing the transverse diameter of the chest wall by the smallest distance between the anterior surface of the vertebral body and the posterior surface of the sternum on axial CT scans of the chest (HI = A/B) [17]. Even though it is not necessary to perform postoperative CT scans in every patient with PC, this was done in our study to acquire optimal pre- and post-surgery chest diameter data (e.g. HI).

The clinical significance of preoperative 3D volume-rendered CT scans lies in the virtual planning of the operation, thus allowing for exact planning of cartilage resection and localization of sternum osteotomies [10].

### Questionnaires

#### International Physical Activity Questionnaire

This questionnaire consists of 27 questions and measures the frequency, duration and intensity of physical activity of the previous week in 4 domains of physical activity (work-related physical activity, transport-related physical activity, domestic and gardening activities and leisure-time physical activity) [21]. It is a reliable and valid tool to assess health-related physical activity in a population aged between 15 and 69 years [21, 22].

#### KGAS—physical activity of adolescents

This questionnaire consists of 13 questions and assess health-related physical activity in a populations aged <15 years. The subscales (School Walking, Outdoor Activity Group, Leisure Activity Group, Leisure Activity Alone) are summed to derive the Total Physical Score. The values reflect the frequency and duration of activity during 1 week.

### Statistical analysis

Data for continuous variables are presented as mean ± standard deviation (SD), and as percentages or frequencies for categorical variables. All parameters were tested for normality using the Kolmogorov–Smirnov test. Paired *t*-tests were performed to compare mean changes in normally distributed variables; the paired Wilcoxon signed ranks test was used for non-normally distributed variables. Absolute differences in the pre–post changes in all variables of interest were calculated with 95% confidence intervals. Statistical significance was accepted at *P*-value <0.05. All calculations were performed using the IBM SPSS statistical software package (version 24.0; IBM Corporation, Armonk, NY, USA).

## RESULTS

For baseline characteristics of patients, refer to Table 1. In all cases, the indication for surgery was psychological. No postoperative complications with surgical revision occurred. All patients had a follow-up of the surgical examinations 8.3 ± 5.4 months (range 4.9–20.7 months) after surgery. All postoperative cardiopulmonary exercise tests were performed 12.9 ± 5.6 months (range 6.1–26.5 months) after the preoperative tests. The present study was limited to functional results. Aesthetic outcomes were not within the scope of this investigation.

**Table 1:** Baseline characteristics of the patients (*n* = 14) pre- and post-surgery

Variables	Pre (mean ± SD)	Post (mean ± SD)
Age (years)	16.2 ± 2.6	17.4 ± 2.5
Body weight (kg)	62.3 ± 10.1	64.4 ± 9.0
Body height (cm)	174.8 ± 11.1	177.7 ± 10.6

Presurgery data were obtained at study inclusion; post-surgery data were measured at the final study examination.

SD: standard deviation.

### Pulmonary function test

The FVC (l), the FEV1 (l) and FEV1/FVC (%) values did not differ significantly between pre- and post-surgery. The FVC (% pred) and FEV1 (% pred) values were significantly lower post-surgery compared to presurgery (*P* = 0.002 and *P* = 0.004, respectively, see Table 2), while FVC/FEV1 (% pred) remained unchanged.

**Table 2:** Data from the pulmonary function tests pre- and post-surgery (*n* = 14)

Variables	Pre (mean ± SD)	Post (mean ± SD)	Δ (CI 95%)	*P*-value (Cohen’s *d*)
FVC (l)	4.9 ± 1.1	4.8 ± 1.0	0.06 (−0.15 to 0.28)	0.55 (−0.16)
FVC (% pred)	102 ± 6.8	94.4 ± 10.4	7.64 (3.42–11.87)	0.002 (−1.48)
FEV1 (l)	4.2 ± 0.8	4.1 ± 0.8	0.05 (−0.13 to 0.24)	0.55 (−0.18)
FEV1 (% pred)	98.9 ±6.7	91.9 ± 10.5	7.00 (2.67–11.34)	0.004 (−1.35)
FEV1/FVC (%)	84.9 ± 5.6	84.7 ± 6.6	0.14 (−1.41 to 1.69)	0.85 (−0.07)
FEV1/FVC (% pred)	96.6 ± 5.6	96.8 ± 6.8	−0.21 (−1.90 to 1.47)	0.79 (0.09)

Data are mean ± SD. *P*-values <0.05 were considered statistically significant.

CI: confidence interval; FEV1: forced expiratory volume in 1 s; FVC: forced vital capacity; % pred: percent predicted value according to Quanjer *et al.* [18]; Pre: baseline (before surgical intervention); Post: after surgical intervention; SD: standard deviation.

### Echocardiography

The end-diastolic diameter at baseline was 34.2 ± 4.4 mm for the right ventricle end-diastolic diameter and 48.2 ± 4.7 mm for the left ventricle end-diastolic diameter. Post-surgery, no significant differences were observed compared to presurgery (right ventricle end-diastolic diameter 33.5 ± 1.8 mm; left ventricle end-diastolic diameter 47.0 ± 1.8 mm).

### Spiroergometry

Results from both cycle spiroergometry tests (sitting and supine position) are shown in Table 3. Only the peak heart rate (bpm) in the sitting position was significantly lower post-surgery compared to presurgery (*P* = 0.02).

**Table 3:** Results from spiroergometry in the sitting and supine positions (*n* = 14)

Variables	Sitting position				Supine position			
	Pre (mean ± SD) (range)	Post (mean ± SD) (range)	Δ (CI 95%)	*P*-value	Pre (mean ± SD) (range)	Post (mean ± SD) (range)	Δ (CI 95%)	*P*-value
Mean power 2 mmol (W/kg)	1.5 ± 0.5^a^ (0.52–2.54)	1.6 ± 0.4^b^ (1.13–2.45)	−0.12 (−0.35 to 0.12)	0.29	1.6 ± 0.4^a^ (0.38–2.28)	1.5 ± 0.3 (0.38–1.9)	0.08 (−0.07 to 0.24)	0.27
Mean power 4 mmol (W/kg)	2.2 ± 0.6 (1.04–3.24)	2.1 ± 0.7 (0.68–3.09)	0.09 (−0.17 to 0.34)	0.47	2.2 ± 0.5^c^ (1.20–2.89)	2.0 ± 0.5 (0.9–2.43)	0.05 (−0.13 to 0.23)	0.52
Peak power (W/kg)	3.3 ± 0.8 (1.34–4.35)	3.2 ± 0.9 (1.03–4.31)	0.17 (−0.14 to 0.47)	0.26	2.6 ± 0.6^c^ (1.32–3.48)	2.5 ± 0.6 (1.05–3.22)	−0.12 (−0.51 to 0.28)	0.52
Peak power (W)	205.5 ± 50.8 (102–275)	203.6 ± 64.5 (77–288)	1.86 (−14.78 to 18.49)	0.81	160.2 ± 45.7^c^ (100–231)	159.4 ± 44.4 (79–208)	−12.4 (−37.65 to 12.85)	0.30
VO_2 peak_ (ml/kg/min)	46.3 ± 9.8 (23.0–59.1)	44.1 ± 11.3 (18.1–55.1)	2.19 (−2.21 to 6.60)	0.30	36.2 ± 8.6^c^ (19.2–47)	35.2 ± 8.6 (17.6–46.1)	−1.53 (−6.73 to 3.67)	0.52
HR_peak_ (bpm)	196.9 ± 8.2 (181–210)	188.6 ± 13.7 (152–210)	8.21 (−0.53 to 16.95)	0.02^b^	170 ± 13.3^c^ (146–193)	172.5 ± 16.6 (146–200)	−3.7 (−12.2 to 4.77)	0.35
Lactate_peak_ (mmol/l)	10.8 ± 1.6 (6.9–13.7)	9.72 ± 2.6 (5.5–13.9)	1.02 (−0.58 to 2.62)	0.19	6.7 ± 1.7^c^ (4.5–9.5)	7.7 ± 2.3 (4.6–10.8)	2.26 (−2.88 to 0.36)	0.11

a *n* = 12.

b Paired Wilcoxon signed ranks test.

c *n* = 10.

CI: confidence interval; HR_peak_: heart rate (peak); SD: standard deviation; Pre: baseline (before surgical intervention); Post: after surgical intervention; VO_2_: oxygen consumption.

### Computed tomography examination and thorax calliper measurements

The mean HI was 1.97 (range 1.6–2.7, SD ± 0.38) and 2.16 (range 1.79–2.8, SD ± 0.30) pre- and post-surgery, respectively (*P* = 0.04).

The mean sagittal chest diameter, as measured by the thorax calliper, showed a reduction from 21.5 cm (range 18–27 cm, SD ± 2.6 cm) to 18.2 cm (range 17–22.5 cm, SD ± 1.8 cm; *P* = 0.002). The mean lateral chest diameter changed from 25.9 cm (range 24–28 cm, SD ± 1.7 cm) to 26.0 cm (range 23–29 cm, SD ± 1.7 cm) (*P* = 0.65).

### Questionnaires

Neither the International Physical Activity Questionnaire nor KGAS data showed significant differences between pre- and post-surgery, for any of the scales.

## DISCUSSION

To address the limitations of previous studies [12], 14 patients were included in our prospective study comparing cardiopulmonary performance parameters pre- and post-surgery in a controlled setting. Our data demonstrated no significant change in cardiopulmonary function during spiroergometry (sitting and supine position) after surgical correction, although there was a reduction in chest diameter.

It is comprehensible that surgical, or even conservative correction in PC patients decreases the intrathoracic volume, thus reducing the space available for the intrathoracic organs. Most patients with PC do not suffer from any clinical symptoms or deficits even during physical exercise, but rather from psychological limitations (marked shyness, ambivalent social behaviour or other psychological disorders due to disturbed body perception). Whether any reduction in intrathoracic volume also reduces exercise performance under different body positions is still a matter of debate. Due to the fixed nature of the anterior-to-posterior chest diameter, respiratory excursions are considerably reduced in PC, followed by an increase in residual air, reduced vital capacity and restricted gas exchange, in turn causing dyspnoea, tachypnoea and reduced exercise performance [16]. Controversy remains, however, regarding the association between PC and respiratory dysfunction, because the number of studies and reported cases is small [13, 23].

Although most institutions perform a thorough evaluation of the patient, unfortunately few papers have reported full data on postinterventional spirometry and exercise performance [24–26]*.* Moreover, follow-up examinations were mainly performed as clinical evaluations, or via telephone or retrospective mail questionnaires, thus not providing objective data [25].

On comparing our results with the existing literature, the following issues were identified. Similar to our study, Cahill *et al.* [13] reported no changes in postoperative pulmonary function test results or progressive work exercise performance in for 5 PC patients after operative repair. In the study of Bagheri *et al.* [15] (*n* = 13), the preoperative evaluation consisted of chest radiography, a CT scan, spirometry and echocardiography. In line with our study, postoperative spirometric measurements did not differ from the baseline measurements [15]. However, Bagheri *et al.* did not report on exercise performance. Derveaux *et al.* [14] included 13 PC patients, for whom lung function evaluations and radiology data were available. Unfortunately, postoperative data were only reported for 7 patients, and the exact time of the postoperative evaluation was not specified. Similar to our PC patients, there were no reductions in FEV1 or FVC pre-compared to post-surgery. In summation of the existing literature, no study definitively showed either significant or clinically relevant changes in parameters of cardiopulmonary function [13–15].

There is conjecture as to whether the body position has any impact on exercise performance in patients with pectus deformities. In contrast to pectus excavatum deformities [27], studies comparing different body posture during stepwise cycle ergometry are missing for PC. Thus, to rule out any effect of body position on exercise performance, stepwise cycle ergometry was done in both the sitting and supine positions. Our results indicate that submaximal and peak power output did not change in either position after surgery. Therefore, we conclude that changes in thoracic geometry do not affect post-surgical submaximal exercise performance.

The exercise programme used over the whole observation period is important when assessing the exercise performance of the patient; changes in exercise habits over several months can affect outcomes. Documenting the patient’s habitual exercise history is an important aspect of the evaluation process. A limitation of several previous studies was that they failed to assess the consistency of physical training, or note relevant changes in exercise habits; in contrast, we examined the physical activity behaviour of all patients. The questionnaire data indicated no significant alterations in the frequency or duration of physical activity postoperatively. Therefore, we speculate that the lack of change in cardiopulmonary performance was not related to changes in physical activity after the surgical intervention, indicating that patients probably did not implement major activity-related life-style modifications.

In our study, postoperative CT scans and calliper measurements showed a reduction in sagittal chest diameter. However, the post-surgical exercise performance was obviously not affected. The only significantly reduced (in terms of % pred values) spirometry parameters were observed for the predictive values of FVC and FEV1. As both of these parameters remained above 90% of an age-matched control group after surgery, no clinically relevant changes in spirometry were observed.

### Controversy regarding the cardiopulmonary benefits of pectus carinatum correction

Some physicians have indicated that there is no correlation between PC and cardiorespiratory dysfunction and that surgical repair should thus be considered primarily for cosmetic benefits [23]. However, Fonkalsrud [2] stated that the high rate of respiratory symptom alleviation, increased exercise tolerance and endurance and improved cosmetic appearance support the view that symptomatic patients with PC of all ages may benefit from repair. Furthermore, the results have been inconsistent, which has complicated the decision of whether to correct PC deformities. Our findings indicated that the surgical intervention did not influence pulmonary or echocardiographic parameters at rest, nor change submaximal exercise performance in the sitting or supine position.

### Limitations

Our study had some limitations. First, selection bias may be inherent in the acquisition of a large population suffering from PC deformities where, due to the long study period and multiple tests, only motivated patients performed all of the postoperative tests, including CT scans and cardiopulmonary function tests. One limitation of all studies involving exhaustive exercise tests is the multifaceted factor of patient motivation. Therefore, the submaximal power at fixed lactate thresholds, rather than the peak, was assessed. A further limitation was that both questionnaires evaluating exercise behaviour only covered the week prior to the test days; no information about exercise behaviour over a longer period (e.g. the last month) is available, pre- or post-surgery.

## CONCLUSION

In conclusion, our findings indicate that surgical correction of PC does not impair cardiopulmonary function at rest or during exercise. Therefore, no adverse effects of surgical treatment of PC on submaximal or even peak exercise performance should be expected.

## ABBREVIATIONS

% pred: Percent predicted values
3D: 3-Dimensional
CT: Computed tomography
FEV1: Forced expiratory volume in 1 s
FVC: Forced vital capacity
HI: Haller Index
PC: Pectus carinatum
SD: Standard deviation
